# Modelling the Innate Immune Response against Avian Influenza Virus in Chicken

**DOI:** 10.1371/journal.pone.0157816

**Published:** 2016-06-21

**Authors:** T. J. Hagenaars, E. A. J. Fischer, C. A. Jansen, J. M. J. Rebel, D. Spekreijse, L. Vervelde, J. A. Backer, M. C. M. de Jong, A. P. Koets

**Affiliations:** 1 Central Veterinary Institute, part of Wageningen UR, Lelystad, The Netherlands; 2 Department of Infectious Diseases and Immunology, Faculty of Veterinary Medicine, Utrecht University, Utrecht, The Netherlands; 3 Department of Farm Animal Health, Faculty of Veterinary Medicine, Utrecht University, Utrecht, The Netherlands; 4 Quantitative Veterinary Epidemiology, Wageningen University, Wageningen, The Netherlands; The University of Chicago, UNITED STATES

## Abstract

At present there is limited understanding of the host immune response to (low pathogenic) avian influenza virus infections in poultry. Here we develop a mathematical model for the innate immune response to avian influenza virus in chicken lung, describing the dynamics of viral load, interferon-α, -β and -γ, lung (i.e. pulmonary) cells and Natural Killer cells. We use recent results from experimentally infected chickens to validate some of the model predictions. The model includes an initial exponential increase of the viral load, which we show to be consistent with experimental data. Using this exponential growth model we show that the duration until a given viral load is reached in experiments with different inoculation doses is consistent with a model assuming a linear relationship between initial viral load and inoculation dose. Subsequent to the exponential-growth phase, the model results show a decline in viral load caused by both target-cell limitation as well as the innate immune response. The model results suggest that the temporal viral load pattern in the lungs displayed in experimental data cannot be explained by target-cell limitation alone. For biologically plausible parameter values the model is able to qualitatively match to data on viral load in chicken lungs up until approximately 4 days post infection. Comparison of model predictions with data on CD107-mediated degranulation of Natural Killer cells yields some discrepancy also for earlier days post infection.

## Introduction

Avian Influenza (AI) in poultry is an important infectious disease as it causes very high economic losses in the poultry industry worldwide and constitutes a zoonotic risk. Its economic importance is evident from the 1999/2000 epidemic of high-pathogenic H7N1 In Italy [1] and the 2003 high-pathogenic H7N7 epidemic in The Netherlands [2, 3]. In Italy over 13 million birds were culled and in The Netherlands 30 million were culled before ‘freedom of disease’-status and access to export markets was regained. Generally, AI epidemics in animal reservoirs in contact with people are considered to pose a risk of emergence of a human pandemic influenza outbreak. The zoonotic risk associated with high-pathogenic avian influenza (HPAI) in poultry is most clearly apparent from the human H5N1 cases in South-East Asia [4], which had a high case fatality rate.

Mathematical modelling of the immune response for influenza in chicken aims to help to interpret observed temporal patterns in viral load and in various immune cell populations, and can be used to formulate and test hypotheses. The modelling carried out here concentrates on the early innate immune responses, including the dynamics of type-I interferons (interferon-α and interferon-β), type-II interferons (interferon-γ), and of Natural Killer (NK) cells. This could serve as a starting point for models that include the adaptive immune responses. Developing model descriptions of viral and innate immune cell dynamics in the host is relevant in particular to on-going efforts to understand the difference in pathology between HPAI and low-pathogenic avian influenza (LPAI) in poultry [5]. In humans and other mammals, high viral load and hypercytokinemia (referred to as cytokine storm [6]) has been found to be associated with fatal outcomes of influenza A (H5N1) infections [6]. If a cytokine storm is also associated with HPAI and fatalities in chickens remains unclear and might depend on the virus strain [7]. It also remains unclear whether or not the difference between LPAI and HPAI pathology is due to the possible existence of differences in NK-cell activating capacity between LPAI and HPAI viruses [8].

Until now, models for the immune response to influenza A virus (IAV) infection have only been developed for the human host [9–13]. For foot-and-mouth disease in pigs and cattle a model has been developed [14, 15], as well as for Salmonella in the chicken intestine [16]. In these papers the models help to explore and test hypotheses about the role of different immune cells in the host response against infection. Baccam et al. [9] show that the natural limitation of host target cells available for the virus may explain the early dynamics of the viral load to a great extent, in particular the occurrence of a sharp drop of viral load after an initial rise. Chang and Young [11] explore the generic behaviour (i.e. across a range of parameter values) of their model of IAV in humans to derive how the time of the initial rise in viral load, the duration and the severity of the infection scale with the number of virus particles present immediately after infection. Also, suitably parameterized models may be used to help estimating the efficacies of potential interventions such as vaccination and antiviral therapy [9].

In this paper we develop a mathematical model for the innate immune responses in the lung to AI virus (AIV) in poultry in order to help interpreting observed temporal patterns in various immune cell populations and viral load dynamics. The data and observations on temporal patterns have been derived from three previously published challenge studies in which chickens were experimentally challenged with HPAI and LPAI strains [5,8,17]. The model development is presented in the Methods section. In the Results section we present the results of three comparisons of the model behaviour to data on viral load and the immune responses in chickens inoculated with AIV. Due to the limitations of the data available for AIV in chicken lungs, we will set some model parameter values using parameter value ranges estimated for IAV in humans [10].

## Methods

The mathematical model we develop is a differential equation model, in part inspired by ideas stemming from the published immune response models in other hosts, mostly in humans [9–13]. Healthy and infected pulmonary cells, and virus, interferon molecules and NK cells in the lungs are the variables whose dynamics are linked by the equations. Also a class of interferon-exposed pulmonary cells is included, which is to some degree protected against infection. The full details of the model are given under “Model development” below.

To estimate model parameters and to validate some of the model predictions we use results of three experimental studies: a study of (immunological host responses and) viral RNA in the lung within the first 24 hours post infection [5], a study of the dependence of infectiousness on inoculation dose [17], and a study of viral RNA and degranulation of NK cells (as a proxy for NK-cell activation) until six days after infection [8]. These datasets are described under “Study data” below.

The approaches used in our model parameter estimation and model validation to the study data are described below in the paragraph entitled “Comparison of model dynamics to data”.

### Model development

#### A model for viral dynamics and the innate response

**Fig 1:**
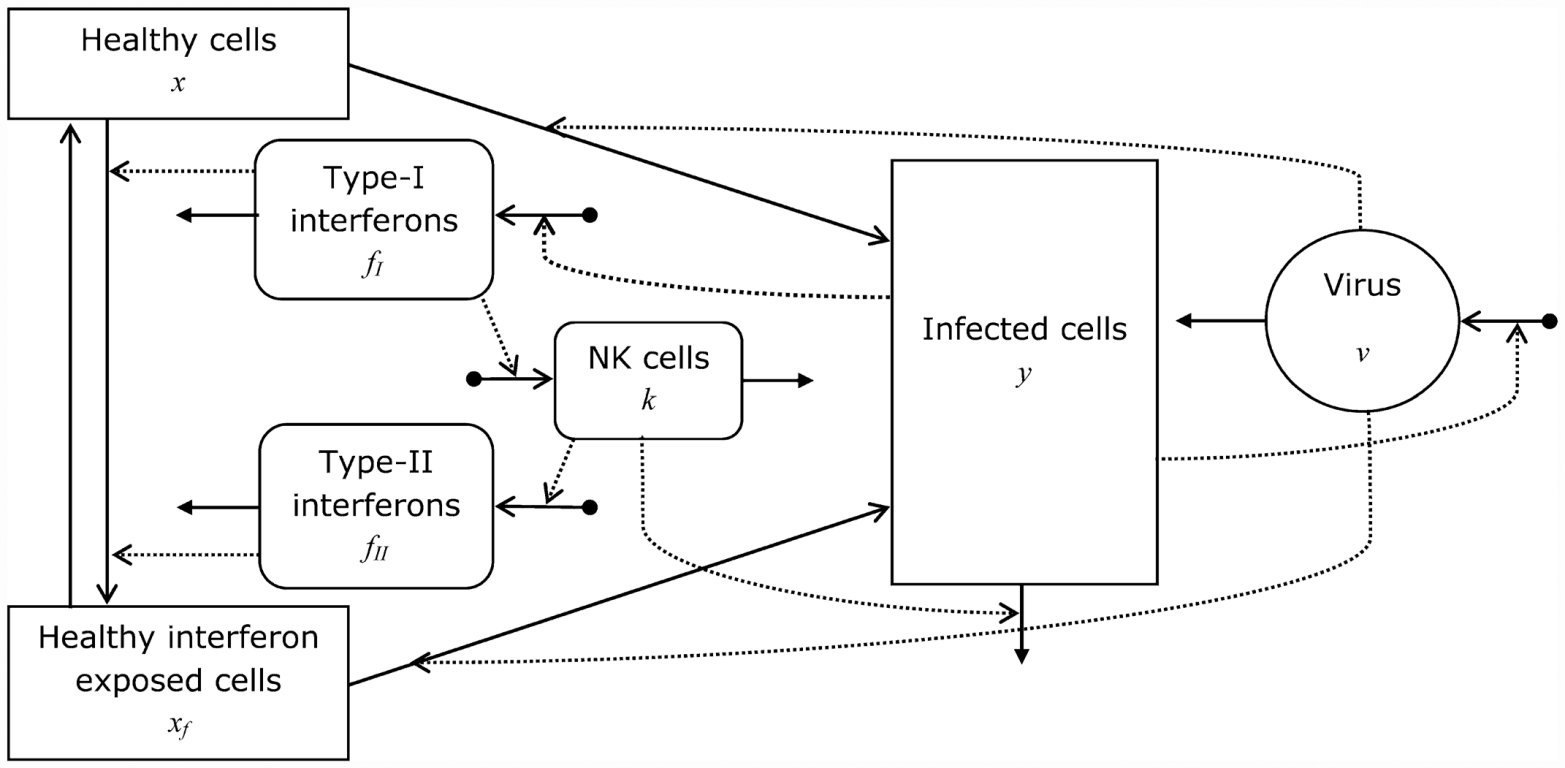
Model structure. Diagrammatic representation of the model, featuring pulmonary cells, virus, interferons and (non-resident) NK cells. Full-line arrows with v-shaped head denote flows between model compartments, corresponding to cell transition rates. Dotted arrows denote “influences” on transition rates. Out-flows from compartments indicated by full-line arrows with triangular head: mortality. In-flows into compartments indicated by full-line arrows with bullet-shaped base: production.

Our model consists of differential equations describing the initial dynamics of the number of virus particles in the lung, *v*, and of the number of uninfected and infected pulmonary cells, denoted by *x* and *y*, respectively. The early immune response is modelled in terms of the number of type-I and type-II interferons *f*_*I*_ and *f*_*II*_, and (non-resident) NK cells *k*. Interferon exposed uninfected pulmonary cells are denoted by *x*_*f*_, and we make no distinction in properties between *infected* cells with and without interferon exposure. A diagrammatic representation of our model is given in Fig 1.

As is indicated in Fig 1 the dynamics of the number of virus particles is assumed to be determined by production of new virus particles in infected cells and by natural decay of virus particles. The corresponding differential equation is:

$$\frac{dv}{dt}=\sigma y-\mu_{v}v.$$

Here, *μ*_*v*_ is the (per-capita) decay rate of viral particles, and *σ* is the production rate of new viral particles from infected pulmonary cells. In line with [9] we here neglect the reduction in viral titer due to binding and infection of target cells, because this accounts for only a very small amount of virus particles.

Assuming that the infection rate is proportional to the number of virus particles and that the rate at which pulmonary cells become interferon exposed is proportional to the number of interferons, yields the following equations for the uninfected, respectively the interferon exposed, uninfected pulmonary cells:

$$\frac{dx}{dt}=-\beta xv-\left(\varphi_{x}^{I}f_{I}+\varphi_{x}^{II}f_{II}\right)x+\alpha x_{f},$$

$$\frac{dx_{f}}{dt}=-\beta_{f}x_{f}v+\left(\varphi_{x}^{I}f_{I}+\varphi_{x}^{II}f_{II}\right)x-\alpha x_{f},.$$

Here, *β* and *β*_*f*_ are infection rate parameters, with *β*_*f*_ < *β* due to the protective effect of interferon exposure, $\varphi_{x}^{I}$ and $\varphi_{x}^{II}$ are the exposure rate parameters, and *α* is the rate of loss of interferon protection for uninfected pulmonary cells.

To incorporate the regeneration of pulmonary cells one may include a logistic growth term in the equation for the uninfected cells. We have found however that adding such a term, when using a similar value of 6.25x10^7^ per day as by Chang and Young [11] for the creation rate of uninfected pulmonary cells (from a pool of precursor cells), has negligible influence on the model dynamics in the first seven days after infection, the period we are interested in here (results not shown).

The influenza A nonstructural protein-1 (NS1) is known to subvert the host interferon response (immune antagonism) [18]. In order to include this mechanism in our model, we subdivide the infected pulmonary cells into two stages:

$$y=y_{1}+y_{2}.$$

Immune antagonism causes the excretion of type-I interferon by an infected cell to be reduced after the virus has substantially replicated in this cell. Therefore we assume that stage 1 is a comparatively short initial stage with a given interferon production, and is followed by a longer stage 2 in which the interferon production is much reduced. When further assuming that the rate of apoptosis of an infected cell induced by NK cells is proportional to the number of NK cells, we arrive at te following equations for the infected cells:

$$\frac{dy_{1}}{dt}=\beta xv+\beta_{f}x_{f}v-\gamma_{y}y_{1}-\mu_{y}y_{1}-\omega_{y}ky_{1},$$

$$\frac{dy_{2}}{dt}=\gamma_{y}y_{1}-\mu_{y}y_{2}-\omega_{y}ky_{2}.$$

Here *γ*_*y*_ is the transition rate of infected cells between stage 1 and 2, *μ*_*y*_ is a mortality rate of infected cells, *ω*_*y*_ is the rate parameter for apoptosis of infected cells induced by NK cells (per infected cell and per NK cell), We assume that the production of type-I interferons by infected pulmonary cells is proportional to the number of infected pulmonary cells:

$$\frac{df_{I}}{dt}=\varphi_{f}^{I}\left(1-\frac{f_{I}}{f_{I}^{*}}\right)(y_{1}+\varepsilon y_{2})-\mu_{f}^{I}f_{I}$$

Here, $\varphi_{f}^{I}$ is the type-I interferon production rate parameter, *f*_I_* is a carrying capacity (which is a constant proportional to the equilibrium number of interferon-producing macrophages [11]), and *ε* is a number much smaller than unity, describing the reduction of interferon production in stage 2, and $\mu_{f}^{I}$ the type-I interferon decay rate. In the model of Baccam et al. [9] the effects of interferons were modelled to occur on the parameters *β* for infection and *σ* for virus production by infected cells directly, i.e. without the use of a structuring in interferon exposed versus non-exposed cells.

The benefit of our description, in which we allow for interferon exposed cells to have a reduced infection rate is that it is more biologically realistic and easier to interpret.

Non-resident NK cells are assumed to be activated by type-I interferons and assumed to produce type-II interferons in proportion to their numbers:

$$\frac{dk}{dt}=\phi_{k}f_{I}-\mu_{k}k$$

$$\frac{df_{II}}{dt}=\phi_{f}^{II}\left(1-\frac{f_{II}}{f_{II}{}^{*}}\right)k-\mu_{f}^{II}f_{II}$$

Here, *φ*_*k*_ is the rate of new non-resident NK cells activated by type-I interferon, $\varphi_{f}^{II}$ is the type-II interferon production rate parameter, *f*_II_* is a carrying capacity and $\mu_{f}^{II}$ the decay rate. We assume that resident NK cells become activated virtually immediately after virus introduction, and denote these by *k*_0_.

We note that as we are considering immunologically naïve chickens, we focus on the innate responses. The effects of the adaptive immune responses are expected to start appearing only towards the end of the period of seven days. To our knowledge, this is the first model that explicitly describes the signalling function of type-I interferons in recruiting activated NK cells, as well as taking into account the production of type-II interferon by the NK cells. In the earlier work on the response in humans, in part due to the importance of the response of cytotoxic T-lymphocytes there, the NK cells, if at all, were included in a cruder fashion as one member of a class of “effector cells” [12].

#### Basic reproduction number and initial increase in viral load

For small initial viral load and low numbers of infected target cells, the equations for *v* and *y* = *y*_1_ + *y*_2_ reduce in linear approximation to:

$$\frac{dv}{dt}=\sigma y-\mu_{v}v$$ (1a)

$$\frac{dy}{dt}=\beta x_{0}v-\mu_{y}y$$ (1b)

From this it can be seen that in this regime the expected number of target cells infected by a single virus particle is *βx*_0_/*μ*_*v*_, and the expected number of virus particles produced by a single infected cell is *σ/μ*_*y*_. The product of these two numbers is the basic reproduction number for within-host viral growth, which is thus given by

$$R_{0}^{\text{wh}}=\frac{\beta x_{0}\sigma }{\mu_{v}\mu_{y}}$$ (2)

with *x*_0_ the initial number of target cells. It can also be derived from Eq (1) that initially the model predicts that the viral load grows exponentially in time,

$$v\left(t\right)=v_{0}e^{\rho t},$$

with growth parameter *ρ* given by function of the five parameters *β*, *x*_0_, *σ*, *μ*_*v*_ and *μ*_*y*_:

$$\rho =\left(R_{0}^{\text{wh}}-1\right)Z\left(R_{0}^{\text{wh}},\mu_{v},\mu_{y}\right)$$ (3)

with $Z\left(R_{0}^{\text{wh}},\mu_{v},\mu_{y}\right)$ a rate defined by a somewhat complicated expression depending on $R_{0}^{\text{wh}},\mu_{v},\mu_{y}$ given in S1 Text. The term $Z\left(R_{0}^{\text{wh}},\mu_{v},\mu_{y}\right)$ is strictly positive, therefore $R_{0}^{\text{wh}}$ is above 1 if and only if the virus growth rate *ρ* is larger than 0.

### Study data

#### Study of viral RNA during the first day after infection

This data is from a study published by Rebel and coworkers [5]. In short, two groups of 30 animals each were inoculated at 21 days of age. One group of birds received 2x10^5^ EID50 of the A/chicken/Italy/1067/99 H7N1 LPAI intranasally and intratracheally, the other group of received 2x10^5^ EID50 of the H7N1 A/turkey/Italy/4580/99 HPAI. Six uninfected chickens from each group were used as controls and 6 infected animals were euthanised at 4, 8, 16 and 24 hours post infection (hpi). Apart from other tissues (data not used in this model study), caudal lung was collected for RNA extraction, snap frozen in liquid nitrogen and stored at -70°C until use. The frozen tissue samples were homogenized, RNA was isolated. A one step RT-PCR reaction was performed to detect the matrix gene of the influenza as a measure for tissue viral RNA. For quantification, a standard curve consisting of 10-fold dilutions of A/chicken/Italy/1067/99 H7N1 LPAI virus with a known egg infectious dose in lung tissue homogenate (EID50/gram tissue) was used.

#### Study of dependence on inoculation dose

This data is from a study published by Spekreijse and coworkers [17]. The HPAI virus strain A/turkey/Turkey/1/2005 H5N1 (clade 2.2) was used as challenge strain for inoculation. Two experiments were carried out consecutively, each with 4 groups of 22 chickens. Each group was housed in a separate room. Per group one inoculation dose was used. The experiments were pair transmission experiments where inoculated or seeder animals were placed in contact with susceptible contact animals. As we are interested here in the dose-dependence of within-host dynamics we will focus on the experimental results for the seeder animals only. In experiment 1, the inoculation doses were 10^2^, 10^3^, 10^4^ and 10^5^ EID50. In experiment 2, the range of the treatment groups was narrowed and the doses used were 10^2.5^, 10^3^, 10^3.5^ and 10^4^ EID50 per chicken. In both experiments, the chickens were inoculated with 0.1 ml inoculum applied intra-nasally and 0.1 ml inoculum applied intra-tracheally. Tracheal swabs (and also cloacal swabs, data not used in this model study) were taken daily. The swabs were put in 2 ml of 2.95% Tryptose Phosphatase Broth with 5 x 10^3^ IU of penicillin—sodium and 5 mg/ml streptomycin and stored at -70°C until analysed. RNA was isolated from 200 ml of swab fluid according to the manufacturer’s instructions. RT-PCR was performed to detect the matrix gene of the influenza as a measure for tissue viral RNA, quantified using a standard curve.

#### Study of virus dynamics and NK-cell activation until one week after infection

This data is from a study by Jansen et al. [8]. At three weeks of age, Lohmann Brown chickens were infected with either the LPAI strain H9N2 isolate A/Chicken/United Arab Emirates/99 (and also with the HPAI strains A/turkey/Turkey/1/2005 or A/turkey/England/50-92/1991, data not used in this model study). Virus was diluted in sterile PBS at a concentration of 1x10^6^ EID50/ml and chickens were inoculated with 0.1 ml intranasally and 0.1 ml intratracheally. Between 0 and 6 days post infection (dpi), birds were euthanized daily lung, spleen and blood were collected. NK-cell activation was assayed in cells isolated from lung and blood using a CD107 degranulation assay as described previously (Jansen et al DCI 2010). Frequencies of NK cells were analysed by flow cytometry as described in [19]. Viral load was determined by matrix gene specific real-time quantitative RT-PCR as described by Van der Goot et al. [20]. Relative expression values were normalized against 28S rRNA, as previously described by Kaiser et al. [21]. Mean threshold cycle values (Ct) were determined based on triplicates and results are shown as 45-Ct values, which are calculated by subtracting the experimental Ct value from the max Ct value (for technical reasons a negative RT-PCR result was given a Ct value of 45 [20]).

### Comparison of model dynamics to data

#### Estimation of the initial rate of viral growth

Assuming that the initial copy number of viral RNA is representative of (i.e. a proxy measure for) the viral load *v*(*t*) in the lung, we may use the data from [5] on viral RNA expression through time during the first day after infection to estimate the growth parameter *ρ*. This approach is valid provided that times until 24 h after infection are early enough for the linear approximation of Eq (1) to the model equations as discussed above to be valid; we come back to this condition in the Discussion. The estimation is performed through a least-squares linear fit to the log-transformed viral RNA expression as a function of time.

#### Analysis of dose-dependent t = 24 h data for viral loads

We analyse the data from the study of dose-dependence as follows. We consider the seeder animals that are developing AIV infection, i.e. for which the trachea swab(s) from some observation time onwards is found positive. When the viral load *v*_0_ immediately after inoculation is assumed to be proportional with factor *θ* to the dose *D* used, *v*_0_ = *θD*, and the time of observation *t*_1_ is early enough for the linear approximation to the model equations to be valid, the model prediction for the dose-dependence of the viral load at the observation time *t*_1_ is:

$${\text{log}}_{10}\left(v\left(t_{1}\right)\right)={\text{log}}_{10}\left(\theta De^{\rho t_{1}}\right)={\text{log}}_{10}\left(D\right)+{\text{log}}_{10}\left(\theta e^{\rho t_{1}}\right)$$ (4)

Therefore, the model predicts that the log-scale viral load observed at a fixed *t*_1_ has a linear dependence on log-dose with slope equal to 1. We may therefore validate the dose-dependence predicted by the model by carrying out a linear regression on the experimental data from the study of dependence on inoculation dose [17] and inspecting from the regression results both the statistical quality of the linear dependence model as well as the consistency with a slope equal to 1. We will choose *t*_1_ = 24 h, i.e. use the earliest observation after inoculation, as only these are expected to be early enough for the linear approximation to the model equations to be valid (see also the Discussion). We assume that the viral RNA expression obtained from the trachea swabs is representative of (i.e. a proxy measure for) the viral load in the lung. For those samples for which the PCR was negative we use an imputed value (chosen below a value of 1.5 log EID50/gram as an approximate value for the detection limit), to construct finite results. In order to account for the uncertainty, we carry out the estimation for several choices of the imputed value, namely 1.0 (“best guess”), 0.5 (low end of plausible range) and 1.5 (high end of plausible range). We note that the estimation approach employed in [15] (using the CDF of the lognormal distribution for the negative samples) has the attractive aspect of avoiding imputation. However we do not use that approach here because taking into account the large uncertainty in the detection threshold within that approach would render it far more computationally intensive than can be justified by the gain in accuracy.

#### Determination of model parameter values

In order make a comparison of predicted model dynamics to observed virus and NK-cell dynamics (for a period until 6 dpi) from [8] we need to determine a full set of model parameter values. We do so by using literature values for some parameters and for other parameters by calculating values either from the estimated initial growth rate *ρ* for LPAI or from literature values for related parameters. The actual construction is discussed in the Results section as it builds on model development results presented there.

## Results

### Estimation of viral growth rate of LPAI vs. HPAI from data

**Fig 2:**
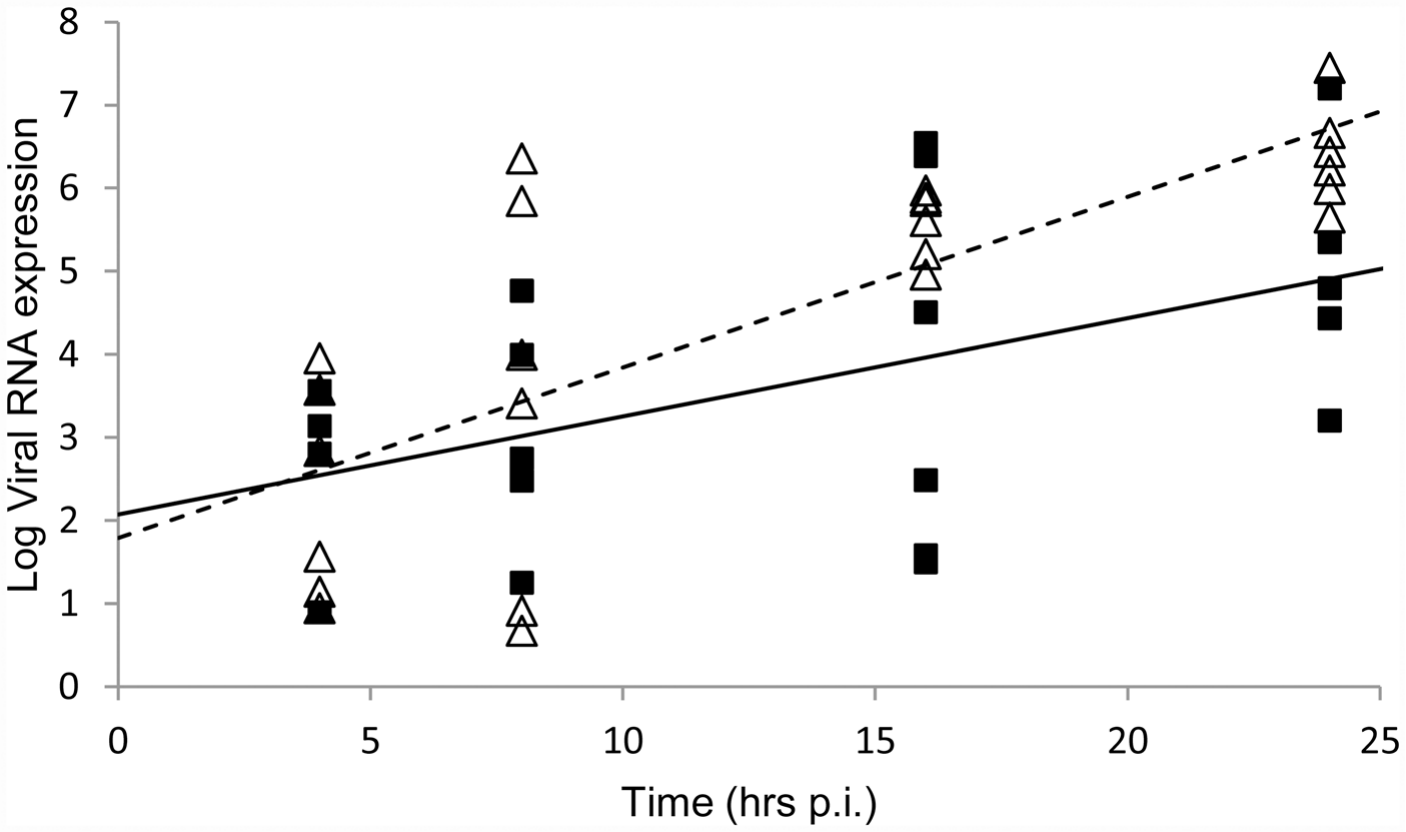
Viral growth estimation. Least-squares linear fits to data from [5] on viral RNA expression (expressed as EID50/gram) in caudal lung tissue through time, estimating the initial exponential growth rate of the number of viral particles. Squares: LPAI, triangles: HPAI. Full line: LPAI fit; dashed line: HPAI fit. Viral RNA expression was obtained from the PCR data as described in the Methods.

In order to validate the prediction of initial exponential growth of the number of viral particles, we consider data on viral RNA expression in lung tissue from Rebel et al. [5]. Our model, being deterministic, describes a mean behaviour, whereas the data shows that there is substantial variation between different samples/animals at any given time point. We estimate the exponential growth rate *ρ* by a least-squares fit to the data on a log-linear scale, thus assuming log-normal variation in the number of viral particles [15]. The resulting linear fits are shown in Fig 2. The fit parameters indicate that the viral load immediately after inoculation (*v*_0_) is similar between the LPAI and HPAI experiments. The estimated initial exponential growth rate *ρ* is higher for HPAI than for LPAI. Although the difference is relevant it is not statistically significant.

Adopting the values *μ*_*v*_ = 2 d^-1^ and *μ*_*y*_ = 1 d^-1^ selected by Chang and Young [11] from the ranges motivated by Bochkarov and Romanyukha [10], we may use the analytical expression given by Eq (3) derived above for *ρ* in terms of *μ*_*v*_, *μ*_*y*_ and $R_{0}^{\text{wh}}$ to calculate $R_{0}^{\text{wh}}$ from *ρ*. Setting *ρ* = 6.5 d^-1^ (11.3 d^-1^), i.e. equal to the LPAI (HPAI) estimate from Table 1, we then find $R_{0}^{\text{wh}}$ = 31.9 (81.9).

**Table 1:** Parameter estimates from the least-squares fits in Fig 2.

Data	*v*_0_	*ρ* (d^-1^)
**LPAI**	119.5 (3.1, 4595.6)	6.5 (0.9, 12.1)
**HPAI**	62.1 (4.4, 886.1)	11.3 (7.1, 15.6)

### Comparison of dose-dependency model to data

**Fig 3:**
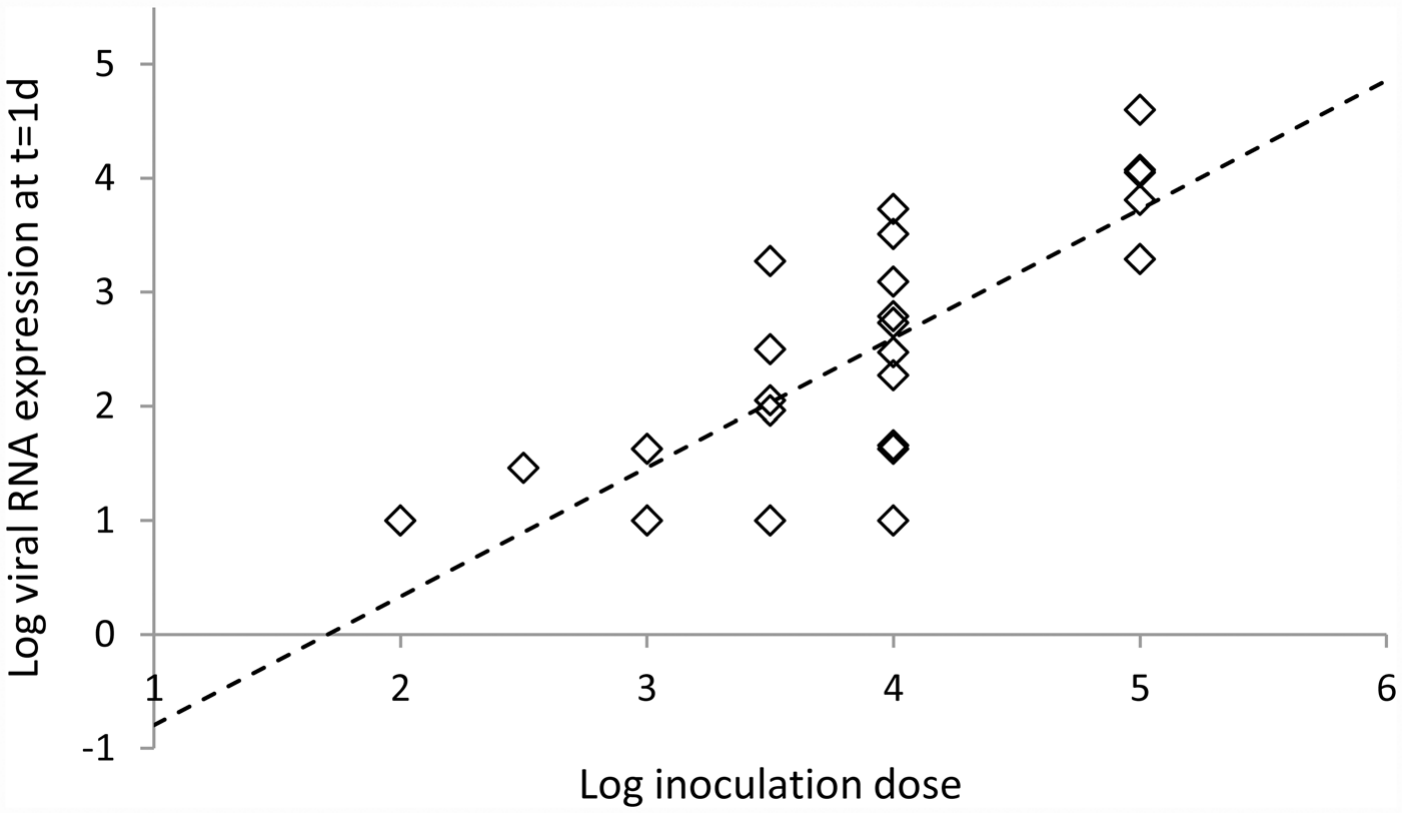
Testing dose-dependency model. Least-squares linear fit (dashed line) to data (symbols) for log viral RNA at t = 24 h after inoculation for different inoculation doses, based on the expected linear dependence Eq (4). Here the imputed value for the negative swabs is 1.0.

In Fig 3 and Table 2 we show the results of the linear regression analyses of the *t* = 24 h data for viral loads from the study of dependence on inoculation dose [17]. The linear dependence of log viral load on log dose predicted by our model yields a satisfactory fit to the data (R Square = 0.64 to 0.66, depending on the imputed value for the negative swabs). In addition, the model prediction that the linear slope has a value of 1 is consistent with the observations: the hypothesis “slope = 1” is not rejected (p>0.05 for all imputed values considered).

**Table 2:** Linear regression results for testing dose-dependency model.

Imputed value	R square	Intercept	Constant (slope)	P value (H0: slope = 1)
0.5	0.65	-2.74 (-4.14, -1.35)	1.38 (0.94–1.61)	0.10
1.0	0.66	-1.94 (-3.11, -0.74)	1.13 (0.81–1.44)	0.40
1.5	0.64	-1.11 (-2.15, -0.07)	0.95 (0.68–1.23)	0.73

Least-squares linear fits to data for log viral RNA at t = 24 h after inoculation for different inoculation doses, based on the expected linear dependence Eq (4), for different imputed values for the negative swabs. Number of observations is 30.

### Comparison of model NK-cell dynamics to data

#### Parameter values

The parameter values chosen are listed in Table 3, and the motivation for these choices is as follows. We set the initial number of uninfected cells *x*_0_ = 10^10^ based on extrapolation from results in [22]. Again using the parameter values *μ*_*v*_ = 2 d^-1^ and *μ*_*y*_ = 1 d^-1^ selected by Chang and Young [11], we set *β* = 1.275x10^-12^ d^-1^ and *σ* = 5x10^3^ d^-1^ (both consistent with the ranges given by [11]) to match (using Eq (2)) the value $R_{0}^{wh}$ = 31.9 and thereby the initial growth rate *ρ* = 6.5 d^-1^ for H7N1 LPAI. For simplicity we now assume that interferon-exposed pulmonary cells are fully protected against infection, i.e. *β*_*f*_ = 0 d^-1^. For the parameters *α* and $\mu_{f}^{I}\left(\mu_{f}^{II}\right)$ we adopt the values chosen in [11], assuming $\mu_{f}^{I}=\mu_{f}^{II}.$ The parameter *ω*_*y*_, the rate of apoptosis of infected cells induced by NK cells, is set at the value 5.0x10^-10^ d^-1^, in line with the upper limit of the plausible range indicated for cytotoxic T-lymphocytes in [11]. The interferon production capacity limits *f*_I_* and *f*_II_* are both set to 1.0x10^9^ d^-1^; we note that inaccuracies in this estimate can be compensated for by adjustment of the parameters *φ*_*k*_,$\varphi_{x}^{I}$ and $\varphi_{x}^{II}$ to match observations on virus and NK-cell dynamics. The production rate $\varphi_{f}^{I}$ of type-I interferon induced by infected cells is set to 1.0x10^4^d^-1^. This order of magnitude is based on multiplying the value of the parameter product Γ_*I*_i* assumed in [11] with *σ*/(*μ*_*v*_ + *ρ*), the virus to infected cells ratio during the initial growth stage. This multiplication factor corrects for the difference in model formulations (the interferon being induced directly by the virions in the model of [11]). We assume that $\varphi_{f}^{II}=\varphi_{f}^{I}$. We set *γ*_*y*_ to 20.0 d^-1^ (fast transition to stage 2) and *ε* to 1x10^-4^ in correspondence with a strong effect of immune antagonism on the interferon production. The values of the five remaining parameters *k*_0_, *φ*_*k*_, *μ*_*k*_,$\varphi_{x}^{I}$ and $\varphi_{x}^{II}$, and the initial number of viral particles (i.e. immediately after inoculation) *v*_0_, are chosen within plausible biological ranges and tuned so as to obtain a model description similar to the experimental data on NK cell and virus dynamics after inoculation with H9N2 LPAI, as discussed in the next paragraph.

**Table 3:** Model parameters, their interpretation and their values used here.

*x*_0_	Equilibrium number of pulmonary target cells	1.0x10^10^
*β*(*β*_*f*_)	Rate parameter of infection of pulmonary cells	1.275x10^-12^ (0.0) d^-1^
*σ*	Release of virus particles from infected cells	5.0x10^3^ d^-1^
*γ*_*y*_	Transition rate of infected cells between stage 1 and 2	20.0 d^-1^
*μ*_*y*_	Death rate of infected cells	1.0 d^-1^
*μ*_*v*_	Decay rate of virus particles	2.0 d^-1^
*α*	Rate of loss of interferon exposure	1.0 d^-1^
*ω*_*y*_	Rate of apoptosis of infected cells induced by NK cells	5.0x10^-8^ d^-1^
$\varphi_{f}^{I}\left(\varphi_{f}^{II}\right)$	Production rate of type-I interferon by infected cells (type-II interferon by NK cells)	1.0x10^4^ (1.0x10^4^) d^-1^
*ε*	Reduction of interferon production in stage-2 infected cells	1.0x10^-4^
$\mu_{f}^{I}\left(\mu_{f}^{II}\right)$	Decay of type-I (type-II) interferon particles	0.5 (0.5) d^-1^
*f*_I_*(*f*_II_*)	Type-I (Type-II) Interferon production capacity limit	1.0x10^9^ (1.0x10^9^) d^-1^
*k*_0_	Resident NK cells (immediately activated upon infection)	2.0x10^5^
*φ*_*k*_	Activation rate of NK cells by interferons	2.0x10^-4^ d^-1^
*μ*_*k*_	Inactivation rate of NK cells	0.8 d^-1^
$\varphi_{x}^{I}\left(\varphi_{x}^{II}\right)$	Protection rate of uninfected cells by type-I (type-II) interferon	1.5x10^-9^ (1.5x10^-9^) d^-1^

#### Experimental and model results until 6 dpi

**Fig 4:**
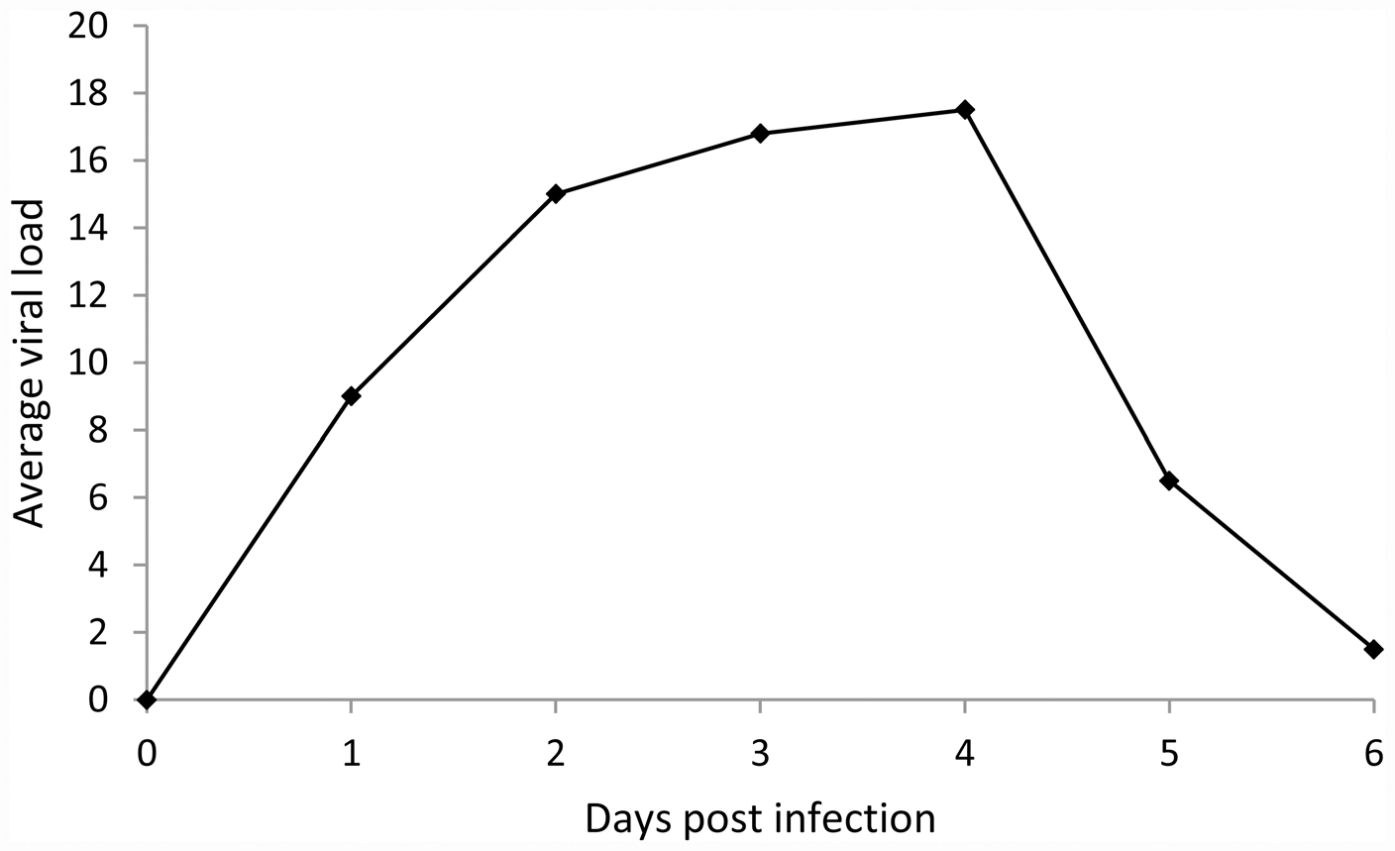
Viral RNA data. Experimental data (dots joined by solid lines) from [8] on H9N2 LPAI viral RNA (45-Ct value of viral RNA) through time until 6 dpi.

**Fig 5:**
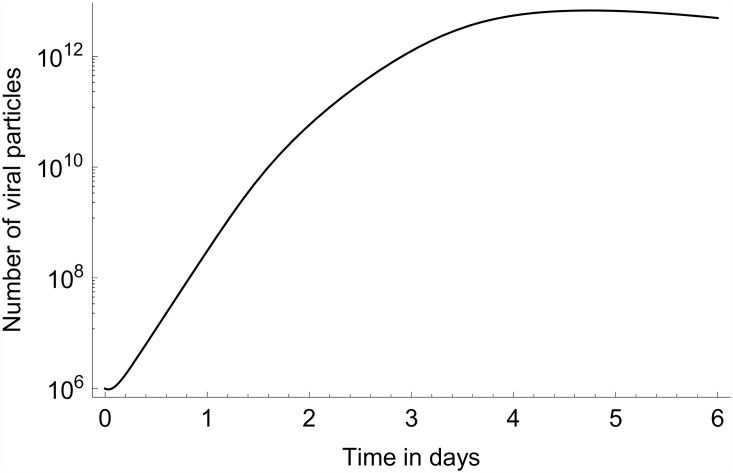
Modelled number of viral particles. Model prediction for the number of viral particles in the lungs using the parameter values of Table 3, and *v*_0_ = 10^6^.

We compare the experimental and model results on viral load in Figs 4 and 5. In this comparison we focus on the time-dependent change of the curves instead of the absolute values. This is because, although both curves are on a logarithmic scale, the scales are different: the model calculates the total number of viral particles in the lungs, whereas the experimental data is expressed as a 45-Ct value for the matrix gene initial copy number extracted from lung tissue samples.

Qualitatively the model produces a satisfactory match to the data up until approximately 4 dpi. For later times the experimental results display a much sharper decline in viral load than the model. This discrepancy indicates that the simplifications used in the model (in particular its focus on interferons and NK-cell responses) might not be appropriate anymore after 4 dpi. A particular feature of the model curve is the (constant rate of) exponential virus growth that extends well past the 24 h time point; this is the case for initial viral load up to *v*_0_ = 10^7^. This shows that for the parameter values used here, the linearization of Eq (1) used above in relating the model to the first two sets of study data is indeed a good approximation to the full model dynamics until 24 h.

**Fig 6:**
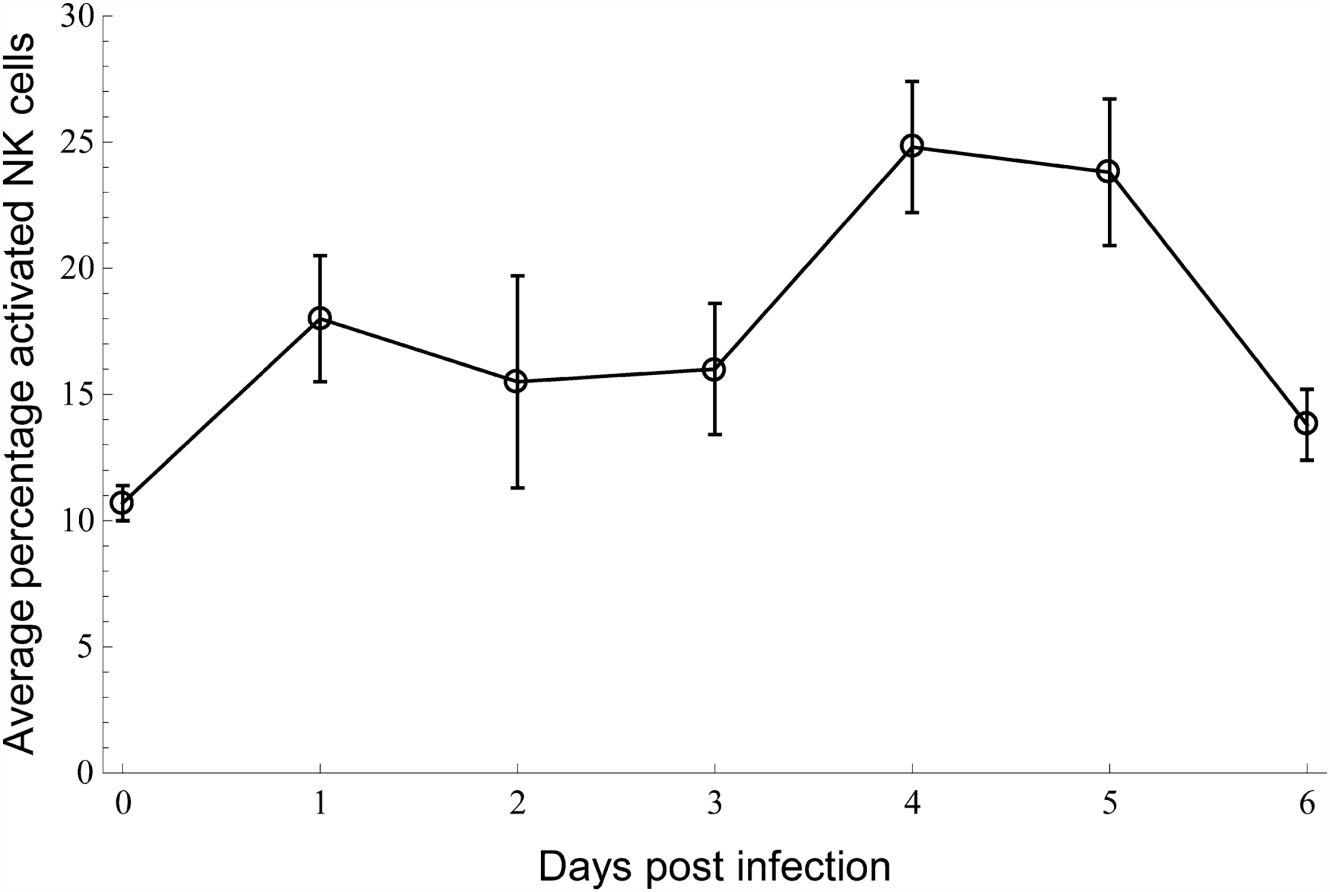
NK-cell activation data. Experimental data (dots joined by solid lines) from [8] for the percentage of degranulated NK cells (CD107 expression) until 6 dpi with H9N2 LPAI.

**Fig 7:**
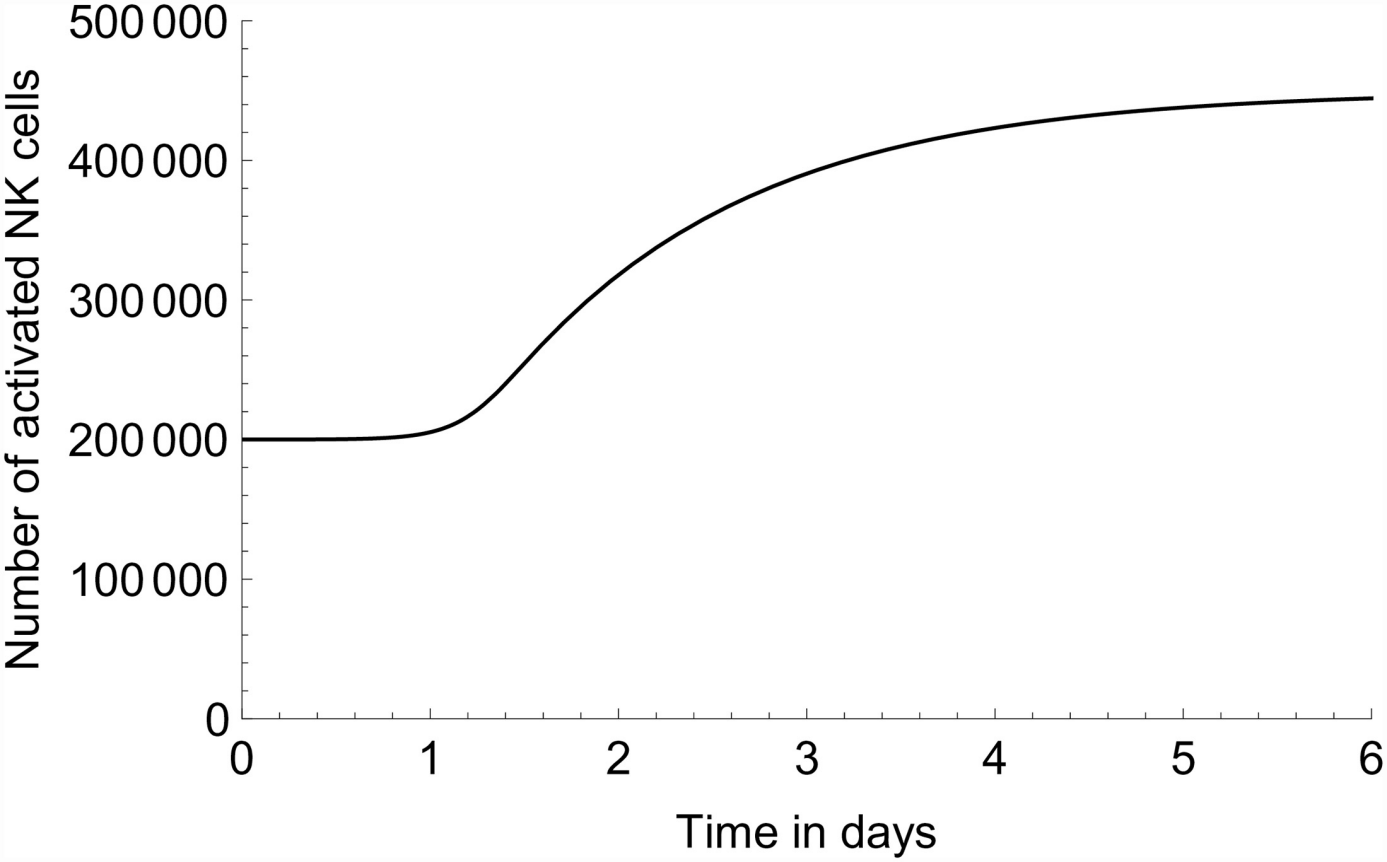
Modelled number of activated NK cells. Model prediction for the number of activated NK cells in the lungs using the parameter values of Table 3, and *v*_0_ = 10^6^.

The results for NK-cell activation are compared in Figs 6 and 7. Also here the model and experimental results are obtained on different scales (here linear), as the model prediction is in terms of total number activated and the experimental results measure the percentage of activated NK cells. The model predicts an increase in activated NK cells up which reaches a plateau at approximately 4 dpi. The experimental results differ from the model prediction in two main respects. Firstly, although showing a (net) increase up until 4 dpi as well, the experimental results are suggestive of a two-step increase. Secondly, after 4 dpi the model and the data diverge, as the latter display a decline that is not present in the model.

**Fig 8:**
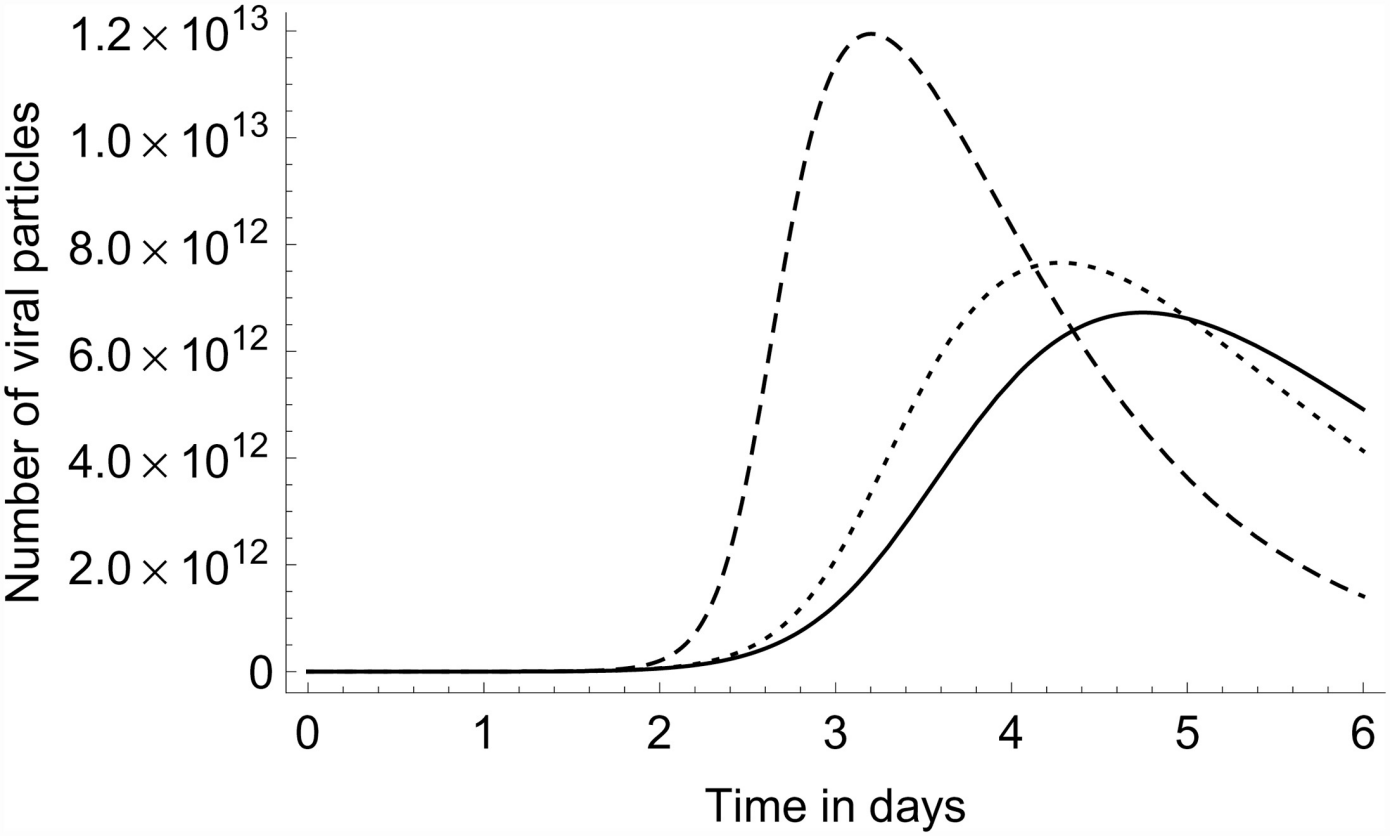
Model truncations. Number of viral particles vs. time. Full line: full model for AIV infection in chicken lung; dotted line: model without NK cells; dashed line: model without interferons, without NK cells. Model parameter values set as in Table 3, and *v*_0_ = 10^6^.

**Fig 9:**
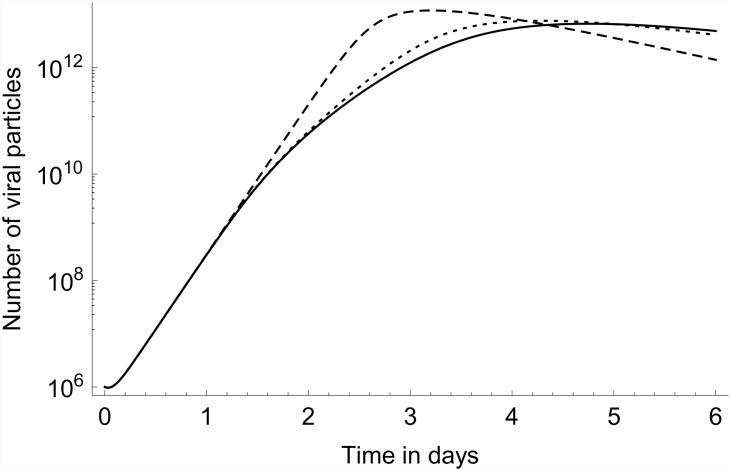
Model truncations. Same as Fig 8 but with number of viral particles on a logarithmic scale.

Relevant to the possible existence of differences in NK-cell activating capacity between LPAI and HPAI viruses [8], we can use the model to investigate the predicted contribution made by the activated NK cells to the decrease in viral load that occurs after the initial increase. In Figs 8 and 9 we compare the predicted full model dynamics to that obtained when truncating the NK cells from the model by setting the parameter *φ*_*k*_ to zero, thus leaving only the interferons response in the model. To put the predicted contribution of the activated NK cells into perspective, we also include the model prediction when both the NK cells and the interferons are truncated from the model. In this latter case the saturation of the growth of the viral particle population is due to target-cell limitation only. As can be seen by comparing the model prediction in Fig 9 for the viral load between approximately 1 and 3 dpi to the experimental data in Fig 4, the temporal viral load pattern in the lungs displayed in the experimental data cannot be explained by target-cell limitation alone: The dampening of the growth in viral load sets in too late if the interferon and NK call responses are switched off in our model. In other words, the data cannot be matched by a model consistent with the data for the first day post infection if target-cell limitation were to be the only mechanism dampening the growth of the viral load.

In addition we observed that the apoptosis of infected cells by the activated NK cells, controlled by the apoptosis rate parameter *ω*_*y*_, has a negligible influence on the model dynamics of the virus and/or infected cell populations, even though the apoptosis rate was set at a value deemed to be at the upper limit of a plausible range. If the parameter *ω*_*y*_ was set to zero, this made no difference to the model results such as shown in Figs 5, 7 and 8.

## Conclusions and Discussion

We have developed a mathematical model for the immune response to influenza virus in the lung of chickens in order to help interpreting observed temporal patterns in NK cell populations and viral load dynamics. The model presents a basis that can be extended with descriptions of the innate response, for example to help evaluating the effects of partial immunity obtained by vaccination or the effects of antiviral therapy [9].

For the initial virus growth until 24 h the model simplifies and only a single (composite) parameter, namely the exponential growth rate *ρ*, determines the essence of the dynamics. We estimated this parameter using least-squares regression. In trying to match the data to experimental data on viral load and NK-cell activation up until 6 dpi, we were interested in establishing whether a qualitative match was possible and not in obtaining detailed parameter estimates. In line with this, we used data for H7N1 to estimate the early exponential growth parameter, although we cannot assume that this parameter value applies in detail to H9N2 for which the data for viral load and NK-cell activation up until 6 dpi were obtained.

- The model developed in this paper is able to describe a number of features observed in experiments: initial virus growth, and subsequent decline due to both target-cell limitation and innate response. Applying the model to the data from three different (sets of) experiments, we obtained the following main insights: Initial virus growth: Experimental data on viral growth in the lung during the first day after infection is consistent with a model with an initially exponential growth.
- Dose dependence: The duration until a given viral load is reached in experiments with different inoculation doses is consistent with a model assuming a linear relationship between initial viral load and inoculation dose.
- For biologically plausible parameter values the model is able to qualitatively match to data on viral load and NK-cell activation in the chicken lung until approximately 4 dpi. For longer times, a poor match to the data indicates that further types of responses not included in the model may become important then.
- The temporal pattern of viral load in the lung found experimentally between roughly 1 and 3 dpi cannot be matched by a model consistent with the data for the first day post infection if target-cell limitation were to be the only mechanism dampening the growth of the viral load.
- We find that the rate of apoptosis of infected cells by the activated NK cells is so small (relative to the virus dynamics) that it has a negligible influence on the dynamics of the virus and/or infected cell populations. This is even though the apoptosis rate per infected cell per activated NK cell was set at a value deemed to be at the upper limit of a plausible range. This finding provides an explanation for the results of experiments in mice showing that cytotoxicity is not necessary for the resolution of infection with influenza [23, 24].

An additional feature that we identified in the model dynamics is the following (results not shown):

- In the model we assumed that activated *resident* NK cells do not induce type-II interferon. If we assume that they do, we find that a satisfactory match of the model prediction to the data of [8] can only be obtained by assuming that the value of the parameter $\varphi_{x}^{II}$ is at least one order of magnitude lower than that of $\varphi_{x}^{I}$, rendering negligible the contribution of type-II interferon to the decrease in viral load. This suggests that to take into account any effect of activated resident NK cells in a realistic manner, an explicit modelling of a delay in the induction of the type-II interferons would be necessary.

Our analysis of the exponential growth phase of H7N1 LPAI in chicken yielded $R_{0}^{\text{wh}}$ = 31.9. We note that this is rougly two times as large as the value that can be calculated for influenza in humans using our expression for $R_{0}^{\text{wh}}$ and the parameter set used in [11] ($R_{0}^{\text{wh}}$ = 17.0). The difference observed in the exponential growth rate of H7N1 LPAI and H7N1 HPAI, although not significant, is relevant. Further studies would be needed to shed light on this potential difference, which might explain part of the difference in virulence.

## Supporting Information

S1 Text Mathematical detail. (DOCX)
