# Supplementary material for: Modelling the Innate Immune Response against Avian Influenza Virus in Chicken
Source: PLoS One. 2016 Jun 21;11(6):e0157816. doi: 10.1371/journal.pone.0157816 (PMC4915690; doi:10.1371/journal.pone.0157816)
Supplement: S1 Text — (DOCX) [file pone.0157816.s001.docx]

**S1 Text. Mathematical detail.**

The rate of exponential growth can be obtained by solving the linearized set of equations. This yields:

$Z\left(R_{0}^{wh},\mu_{v},\mu_{y}\right)=\frac{2\mu_{v}\mu_{y}}{\left(\mu_{v}+\mu_{y}\right)\left(1+\sqrt{1+4\frac{\left(R_{0}^{wh}-1\right)\mu_{v}\mu_{y}}{\left(\mu_{v}+\mu_{y}\right)^{2}}}\right)}$
